# Sociodemographic and Lifestyle Factors in relation to Overweight Defined by BMI and “Normal-Weight Obesity”

**DOI:** 10.1155/2020/2070297

**Published:** 2020-01-07

**Authors:** Bodil Ohlsson, Jonas Manjer

**Affiliations:** ^1^Lund University, Skane University Hospital, Department of Internal Medicine, Malmö, Sweden; ^2^Lund University, Skane University Hospital, Department of Surgery, Malmö, Sweden

## Abstract

Sociodemographic factors and lifestyle habits affect body weight and body composition. A new syndrome, called normal-weight obesity (NWO), is found in individuals with normal weight and excess body fat in contrast to lean and overweight individuals. The aim of the present study was to explore the associations between sociodemographic factors and smoking and alcohol habits and lower versus higher BMI (≥25 kg/m^2^) and to examine whether categorization into lean, NWO, and overweight leads to further information about sociodemographic and lifestyle associations, compared with the common categorization defined by BMI. A cohort of 17,724 participants (9,936 females, 56.1%) from the EpiHealth study, with a median age of 61 (53–67) years, was examined. The participants answered a questionnaire about lifestyle, and weight and fat percentage were measured. Associations between sociodemographic factors and lifestyle habits and lower versus higher BMI, and lean versus NWO or lean and NWO versus overweight were calculated by binary logistic regression. Male sex, age, sick leave/disability, married/cohabitating, divorced/widowed, former smoking, and a high alcohol consumption were associated with higher BMI, whereas higher education and frequent alcohol consumption were inversely associated (all *p* < 0.001). The associations were similar to associations with lean versus overweight and NWO versus overweight, except for age in the latter case. Associations with lean versus NWO differed from those of lower versus higher BMI, with an association with retirement, an inverse association with male sex (OR, 0.664; 95% confidence interval, 0.591–0.746), and no associations with marital status, smoking, and alcohol consumption frequency. Associations with age and occupation were sex dependent, in contrast to other variables examined. Thus, sociodemographic and lifestyle habits showed similar associations with lower versus higher BMI as with lean and NWO versus overweight, whereas lean versus NWO showed different directions of associations regarding sex, marital status, occupation, smoking, and frequency of alcohol consumption.

## 1. Introduction

Altered lifestyle habits and improved socioeconomic conditions may explain why weight and body mass index (BMI) are increasing in the Western world [1]. Not only the total amount of fat mass but also its distribution is important for health. Adipose tissue is mainly deposited as subcutaneous adipose tissue and intra-abdominal adipose tissue, but fat may also be deposited as epicardial adipose tissue and intermuscular adipose tissue [2–4]. The visceral (abdominal) fat is more closely associated with metabolic disorders than subcutaneous fat [2, 3]. A new syndrome, called normal-weight obesity (NWO), is found in subjects with a normal BMI of <25 kg/m^2^ but with excess body fat [5, 6]. A disadvantage with BMI is that BMI only measures weight in relation to height, without adjustments for the distribution between muscle and adipose tissue, which may be of importance for health. Previous studies have shown that low physical activity, ex-smoking, and alcohol consumption were associated with NWO, rather than with lean and overweight [6], and that NWO shows a high degree of metabolic dysregulation, in spite of normal BMI [7]. NWO may be present in all ages, from childhood to adolescence and adult subjects, and it is associated with higher risk for poor skeletal robustness, metabolic dysregulation, and development of the metabolic syndrome [8, 9].

There is a lot of debate and controversy about the most efficient lifestyle habits for maintaining a low body weight throughout life. Although all fat deposits are associated with age [4], most studies are performed on younger individuals [10]. Education, income level, physical activity, smoking, and alcohol consumption have been shown to be related to the percentage of energy intake from different macronutrients [10–12]. In addition to the influence on dietary habits, smoking also affects the secretion and regulation of hormone levels [13]. Alcohol contains more energy per gram (7 kcal/g) than carbohydrates and protein (4 kcal/g) and slightly less energy than fat (9 kcal/g) [14]. Because alcohol does not replace other food intakes but instead is added to the diet, alcohol consumption may lead to weight gain [12].

Recent research has shown that the middle-aged and elder population has a high alcohol consumption, above what is considered to be healthy [15]. On the other hand, many people of that age range do not longer smoke. Overall, lifestyle habits have changed markedly during the past decades and are more equalized between sexes than in previous generations, which influence body weight and body composition [16]. The Northern Sweden MONICA project, performed from 1986 to 2004, has shown how BMI has markedly increased over time [17]. Thus, former sex differences and associations with sociodemographic and lifestyle factors may have changed, as well as the relation between body weight and fat percentage. To address these questions in relation to elder subjects, the Swedish EpiHealth cohort of 17,724 participants between the ages of 45 and 75 years was examined. This cohort has previously been used to study the effect of physical activity, irregular meals, and BMI on gastrointestinal symptoms [18]. The aim of the present cross-sectional study was to explore the associations between sociodemographic factors and smoking and alcohol habits and (1) lower versus higher BMI, (2) lean versus NWO, and (3) lean and NWO versus overweight. Second, we wanted to examine whether categorization into lean, NWO, and overweight leads to further information about sociodemographic and lifestyle associations, compared with the common categorization into lower and higher BMI.

## 2. Methods

The study was performed in accordance with the Declaration of Helsinki. The original EpiHealth study was approved by the Ethics Review Board of Uppsala University (2010/402) and by the Swedish Data Inspection Board (307-2011). All participants gave their informed written consent before inclusion. The present part of the study was approved by the Ethics Review Board at the Lund University (2014/4), along with a separate application to the steering group of EpiHealth. According to the instructions given to the participants upon enrollment [18], an advertisement was published in daily newspapers, where the participants included were encouraged to contact the manager of the study if they did not want to participate in the actual calculations. In addition, we had to display a short description of the research proposal on EpiHealth's homepage for 1 month, so that participants could withdraw consent if they were not comfortable with the current research proposal. No one contacted us to prohibit the use of the data.

### 2.1. Subject Recruitment

A methodological description of EpiHealth has previously been published [19]. Briefly, EpiHealth is a collaboration between the Lund University and the Uppsala University, aiming to build a Swedish resource of a multicenter longitudinal cohort of 300,000 individuals. The EpiHealth study includes three parts: a web-based baseline questionnaire completed by the participants, physical tests and biological sampling performed at a test center, and possibilities to follow-up later on concerning future diseases and medications, through official Swedish registers [19].

All people between the ages of 45 and 75 years with Swedish personal identity number, who live close to the test centers Uppsala or Malmö, are invited to participate in the study. The participants receive no financial compensation. The only exclusion criteria is unwillingness to participate.

An invitation letter was sent to the subjects, along with activation codes and instructions on how to enroll in EpiHealth on the website (http://www.epihealth.se) or via the EpiHealth Screening Center.

About 50,000 inhabitants had been invited to participate in the study, at the time of data extraction. The present participants were enrolled from 2011 to 2014. Thus, the current study cohort represent the first recruits to EpiHealth.

### 2.2. Physical Tests

Upon arrival at the EpiHealth Test Center, the participants were provided with oral and verbal information about the study, before the consent was signed. The participants were asked whether they had had a current infection/inflammation within the last 2 weeks and whether they had eaten during the day. If the participant answered “no,” weight was recorded in light clothes and with an empty bladder at a scale that uses bioimpedance to calculate fat mass (Tanita, Tokyo, Japan) [19].

### 2.3. Data Categorization

The web-based EpiHealth questionnaire included questions about sex, sociodemographic factors, family history, lifestyle habits, medical health, pharmacological treatment, and subjective suffering from pain and discomfort. The questionnaires used in EpiHealth are not validated questionnaires but are similar to questionnaires used in other large population-based screening projects in Sweden (e.g., LifeGene, SCAPIS, and BIG-3). The time to complete the form was 40–60 minutes. For those participants who needed computer assistance, computers were available at the test center. The questionnaire contained detailed questions about smoking, both regarding how many years they had been smoking and the amount of tobacco used daily. Participants were asked about the type of alcohol consumed, the number of standard drinks, and the frequency of drinking. They rated their degree of physical activity from a number of alternatives. The participants answered questions concerning their food intake, in relation to a wide range of different foods, and the amount and frequency of such intake.

Variables concerning age, sex, education, occupation, marital status, tobacco use, and alcohol use were chosen to be studied because from experience, they may influence the development of health, body weight, and body composition and have been changing considerably during the past years [7, 15, 16, 20]. The variables of age and BMI showed skew distribution. Age groups were sorted into 45–49 years, 50–59 years, 60–69 years, and 70–75 years. BMI was grouped into normal-weight (<25 kg/m^2^), overweight (25.0–29.9 kg/m^2^), obese class 1 (30.0–34.9 kg/m^2^), and obese class 2 (≥35.0 kg/m^2^) [21], to describe the population. To calculate associations between BMI and sociodemographic factors and lifestyle habits, BMI was divided into <25 kg/m^2^ and ≥25 kg/m^2^, which is the BMI value used to divide subjects into lean, NWO, and overweight [5, 6]. In addition, the participants were divided into lean individuals, with a BMI of <25 kg/m^2^ and a fat percentage in men of <20% and in women of <30%; individuals with NWO, with a BMI of <25 kg/m^2^ and a fat percentage in men of ≥20% and in women of ≥30%; and overweight individuals, with a BMI of ≥25 kg/m^2^ [5, 6]. Educational level was divided into primary school, secondary school, and higher education. Occupation was divided into working, sick leave/disability, retirement, and others, including unemployed and studying. Marital status was divided into single/living alone, married/cohabitating, and divorced/widowed.

Variables about smoking, alcohol consumption, and physical activity are divided according to previous publications [18, 22]. Smoking habits were categorized into three groups: never smoked (or less than 100 cigarettes in total), former smokers, and current smokers (including irregular smoking). The frequency of alcohol consumption was categorized into: never, ≤1 time per month, 2-3 times per month, once per week, 2-3 times per week, or ≥4 times per week. The amount of standard drinks on a typical day was divided into 1-2 glasses, 3-4 glasses, or ≥5 glasses.

Physical activity during leisure time was estimated by cases in the questionnaire, ranging from mostly sitting, light activity, walking 30 min/day, activity 30–60 min/day to strenuous activity 60 min/day. Physical activity during occupational time was not included in the present study because only half of the participants were working, and a high activity during occupation time correlated with a high activity during leisure time (data not shown). The frequency of meal intake was estimated separately for breakfast, lunch, and dinner. A meal taken at least 5 times per week was considered as a regular meal [18].

### 2.4. Statistical Analyses

The data were analyzed using the software SPSS, version 23.0 for Windows. Results are presented as number and percentages or medians and interquartile ranges (IQR). The influence on BMI and lean/NWO/overweight (dependent variables) of sociodemographic factors and lifestyle habits used (independent variables), namely, age, sex, education, occupation, marital status, smoking habits, alcohol drinking frequency, and the amount of drinking on such occasions were initially examined using an unconditional logistic regression to calculate the odds ratios (OR) and a 95% confidence interval (CI). The reference was set to the lowest category of each variable. Analyses were then performed, adjusted for age, sex, education, occupation, marital status, smoking habits, alcohol drinking frequency, and the amount of drinking on such occasions, in addition to physical activity and the irregularity of breakfast, lunch, and dinner because these parameters have been found to influence BMI and fat depositions in another study [6], and adjusted OR with a 95% CI are presented. An interaction analysis was performed on the sex and each factor in the adjusted model by including an interaction term. Logistic calculations were performed separately for women and men, when statistically significant sex interactions (i) were present. The adjusted analyses were performed as a complete case analysis. A *p* value of <0.05 was considered statistically significant.

## 3. Results

### 3.1. Basic Characteristics

Of the invited subjects, 17,724 subjects (9,936 women, 56.1%) were willing to participate and were included in the EpiHealth study. The median age was 61 (53–67) years. Forty-five percent had a higher education, whereas 25% answered that secondary school was their highest educational level. Fifty-six percent were still working, and 35% were retired. The majority were married or cohabitating. Forty-eight percent of the participants had never smoked, and the rest of the participants were mostly former smokers. Less than 10% were still smokers. The most common alcohol drinking habit was an alcohol consumption of 1-2 glasses, 2-3 times a week (Table 1). Approximately 40% of the participants answered that they had a physical activity during their leisure time corresponding to 30 minutes of walking each day. More than 90% of the participants had regular dietary habits (data not shown).

### 3.2. Body Mass Index and Body Composition

The BMI values in the whole cohort was 25 (23–28) kg/m^2^ from 17,626 valid participants (missing values = 98). The distribution of BMI showed that 40% were categorized as normal-weight, 43.3% were categorized as overweight, 13% were categorized as obese class 1, and 3.2% as obese class 2. When dividing the cohort into three groups—lean, NWO, and overweight—the majority of participants were categorized as overweight (missing values = 251) (Table 2). BMI was lower in the lean group than in the NWO group (22 (20–23) versus 23 (22–24) kg/m^2^; *p* < 0.001).

### 3.3. Sociodemographic and Lifestyle Factors

Male sex, age, being on sick leave/having a disability, being married/cohabitating, being a former smoker, and drinking 3-4 glasses per occasion were associated with higher BMI (all *p* < 0.001), whereas higher education and frequent alcohol consumption were inversely associated with BMI (*p* < 0.001) (Table 1). Being divorced/widowed was also associated with a higher BMI (OR: 1.177; 95% CI: 1.036–1.336; *p*=0.012), whereas only a significant trend was seen in the association between being retired and a higher BMI (*p*=0.060) (Table 1).

Similar associations were found regarding associations with male sex, being on sick leave/having a disability, being married/cohabitating, being a former smoker, and a higher amount of drinking per occasion, and inverse associations were found with having a higher education and frequent alcohol consumption, when comparing lean versus overweight and NWO versus overweight. The differences were that retirement was positively associated, and current smoking was inversely associated, with overweight in comparison to lean, whereas age did not associate when NWO was compared with overweight (Table 3).

Age, being retired, and consuming a larger amount of alcohol per occasion were associated with NWO, whereas male sex and having a higher education were inversely associated with NWO, when comparing lean versus NWO. Marital status, smoking habits, and the frequency of alcohol consumption did not show any associations with fat percentage, as long as the subjects were of a normal-weight (Table 3).

### 3.4. Sex Interaction Aspects

Older, retired, or disabled women were associated with a higher BMI, which was not found in men (Table 4).

Although age was neither significantly associated in women nor men, there was a tendency toward a positive association between age and overweight compared with NWO in women, and a tendency toward a negative association in men (*p*_*i*_=0.001). Being on sick leave/having a disability was associated with overweight in women (*p*_*i*_=0.001), and retirement was inversely associated with overweight in men (*p*_*i*_ < 0.001) (Table 5). Education, marital status, smoking, and alcohol habits did not show any sex interactions, neither regarding higher BMI nor NWO versus overweight (data not shown).

The only significant sex interaction regarding lean and overweight was an association between age and overweight in the age group of 50–59 years, which was observed in women but not in men (OR, 1.870; 95% CI, 1.585–2.206 and OR, 1.163; 95% CI, 0.938–1.443, respectively; *p*_*i*_=0.002), in comparison to lean.

Being retired showed a stronger association with NWO in men than in women, when compared with lean (OR, 1.715; 95% CI, 1.293–2.275 and OR, 1.464; 95% CI, 1.170–1.831, respectively; *p*_*i*_=0.001). No other sex interactions were found between lean and NWO (data not shown).

## 4. Discussion

The main findings of the present study were that male sex, age, being on sick leave/having a disability, being married/cohabitating, being divorced/widowed, being a former smoker, and having a high alcohol consumption per occasion were associated with a higher BMI, whereas a higher education and frequent alcohol drinking were associated with a lower BMI. Similar associations were found with NWO versus overweight, except for age, and with lean versus overweight, except for no association with being divorced/widowed, and a positive association with being retired and an inverse association with being a current smoker. Associations with lean versus NWO differed from associations with BMI, with an inverse association with the male sex, and without associations with marital status, smoking, or frequency of alcohol intake. The associations between higher age and higher BMI or overweight, and between sick leave/having a disability and higher BMI or overweight, were due to associations in women. NWO was associated with retirement, especially in men.

The lower BMI found in participants with higher education, and the higher BMI and overweight found in participants on sick leave/disability, is in line with previous research, which has shown that obesity is associated with sociodemographic factors, especially in women [10, 23, 24]. Overall, a higher neighborhood level and higher socioeconomic status were observed to be associated with a lower BMI, waist circumference, and waist/hip ratio [25]. This may be due to a healthier diet in subjects with a higher level of education, income, and socioeconomic status [12], as well as a higher degree of physical activity [25]. However, the associations have to be interpreted with caution. Selection bias has previously been reported in epidemiological studies, with higher age, higher education level, and higher income among participants than nonparticipants [26]. This selection bias may partly explain some of the associations.

Being married/cohabitating was associated with a higher BMI and being overweight, compared with being single/living alone. Previous research has shown that married men were the least likely to be smokers and consumed more vegetables and cereal fibers than divorced men. On the other hand, divorced men were more physically active, consumed more alcohol, and were more often smokers than men in other marital arrangements [27]. The conclusion is that marital status has a high impact on lifestyle habits, and this is reflected by the altered BMI.

Current smoking was associated with a lean body constitution and tended to be associated with a lower BMI. This may be due to the strong endocrine effects caused by smoking. Smoking has been shown to induce increased levels of cortisol and testosterone, whereas the levels of estradiol and progesterone are decreased [13]. This may influence the body composition, changing it to a more android-type composition. Furthermore, smoking has been reported to change the dietary habits, with a lower intake of fibers, fruits, and vegetables [13]. Smoking cessation leads to great changes in endocrine and metabolic balances, which may explain the increased BMI in ex-smokers [28].

In general, alcohol intake is added to the diet, without a corresponding decrease of energy from food intake, and thereby, the total daily energy intake is increased considerably. Thus, a high alcohol consumption per occasion induces an increase in body weight [10, 12], which was also found in the present study. Furthermore, alcohol stimulates the appetite, causing a higher food intake, along with the alcohol consumption [10]. High energy intake from alcohol was positively associated with intermuscular adipose tissue in older subjects, especially in women [14]. The changed lifestyle habits, with an increased alcohol intake in both sexes may be one of the most important factors for the obesity epidemic observed in the Western world [10, 16]. The findings in the current study may be generalized to other countries as well and not only restricted to Sweden. The present finding of a lower BMI in patients who frequently consume alcohol is in accordance with previous studies, which show that frequent alcohol consumption was associated with a less healthy diet, malnutrition, and weight loss [12, 29]. Overall, drinking patterns are closely associated with socioeconomic status, which may have impact on the present results, and underlines the importance of examining sociodemographic factors and lifestyle habits together [30].

Body fat distribution is described as android (apple shaped) and gynoid (pear shaped) [31]. In postmenopausal women, fat mass increases, and the body composition changes from a gynoid to an android form. There may be several causes of the associations between age and BMI and NWO, e.g., less physical activity or a less healthy diet with increasing age. On the contrary, the present participants were 45–75 years old. In this age range, the older women seem to have a healthier dietary pattern than the younger ones [11]. In accordance with our results, all fat deposits have previously been found to be associated with age [4]. This may be due both to genetic and environmental factors [32, 33]. Changes in lifestyle habits with a decrease in smoking and a higher alcohol consumption in both sexes, along with dietary changes, influence body composition over time [16]. Age, being retired, and a high amount of alcohol consumption per occasion seem important factors in relation to fat percentage and the corresponding reduced muscle mass. The greater association of retirement with NWO in the male than female sex may be due to that men may have more physically strenuous work than women and, perhaps, also because they have less active leisure time than women. Previous results showed that men with NWO had the most physically inactive leisure time, compared with lean and overweight men [6]. On the other hand, marital status, smoking habits, and the frequency of alcohol consumption were not associated with fat percentage in normal-weighted participants. The associations suggest that factors normally associated with a lower degree of physical activity, and thereby decreased muscle mass, are important for the development of the NWO syndrome. The associations with overweight versus lean or NWO were similar but not regarding age. Together, the findings suggest that both a higher fat percentage and heavier weight are common in elder women.

This is the first study, to our knowledge, which compare associations with BMI of sociodemographic factors and smoking and alcohol habits, to associations of these factors with the NWO syndrome. One of the restrictions to the study, which may influence the outcome and interpretation of the results, is that the definitions for NWO are not clearly stated, and several values are given in the literature due to no clear cutoff for the most optimal fat percentage values [7]. We have chosen the actual fat percentage limits because they have been used in several other studies, including one with a Nordic population [6, 7]. However, change of the reference values for fat percentages may affect the outcome.

Because the present study is a cross-sectional study, we do not know whether the status of overweight or NWO have been present during a longer period of the participant's lives or whether the conditions have been developed in the middle age or later. This has to be studied in prospective population studies. Nevertheless, independent of age, it is important to recognize the condition due to the high impact on health [7, 8] and to be aware of factors associated with the condition, as described in the present study, to be able to prevent the development. The different associations with NWO compared with overweight might to some extent depend on smaller cohort size in this group.

The sex interactions in relation to occupations may be due to socioeconomic factors, which still show great differences between men and women [20]. However, there were no sex interactions, in relation to smoking or alcohol habits, concerning BMI or NWO. Previously, an association was found between men with NWO and age and smoking, compared with lean and overweight subjects, whereas women with NWO were associated with being an ex-smoker and having a high alcohol consumption, compared with other groups [6]. These previous gender differences were not found in the present cohort. The differences between the cohorts may depend on, e.g., higher age in our cohort, cultural differences between the countries and different sizes of the cohorts. The absence of sex interactions regarding smoking and alcohol consumption in the current cohort may be explained by the fact that these lifestyle habits are now more similar in men and women than in previous generations [16].

The strength of the present study is the large cohort size and its study of NWO in addition to BMI in elder participants. One limitation of the present study is the lack of information and adjustment of menopausal status because body fat mass is increased after menopause [34], which may have affected some of the calculations. After the inauguration of EpiHealth, a recent publication has shown that bioimpedance measurement is a less reliable method for elder patients, with an underestimation of fat mass [35]. The low inclusion rate remains another limitation, with no nonresponse analysis.

## 5. Conclusion

Both sociodemographic factors and smoking and alcohol habits are associated with weight and body composition in a middle-aged and elder Swedish population. Lower versus higher BMI shows similar associations as lean versus overweight and NWO versus overweight, whereas lean versus NWO differs in associations. The differences between lower versus higher BMI, and lean versus NWO, are that lower versus higher BMI is associated with male sex, sick leave/disability, married/cohabitating, former smoking, and frequent alcohol intake, whereas lean versus NWO is inversely associated with male sex, is associated with retirement instead of sick leave/disability, and show no associations with maternal status, smoking, and alcohol consumption frequency. However, both higher BMI and NWO are associated with increased age and high alcohol consumption at each drinking occasion but inversely associated with higher education. Thus, dividing the cohort into lean, NWO, and overweight individuals provide further information on how sociodemographic factors and lifestyle habits affect body composition than if the cohort is only divided into different BMI groups, irrespective of fat percentage. Although associations with age and occupation differ depending on sex, no sex interactions in this study cohort of elder subjects were shown regarding education, marital status, smoking, and alcohol habits. Because epidemiological studies only describe associations and thereby generate hypotheses, these associations have to be confirmed in clinical trials.

## Figures and Tables

**Table 1:** Associations between BMI and sociodemographic factors and smoking and alcohol habits in the EpiHealth cohort.

	BMI < 25 kg/m^2^*N* = 7,089 (40.0)	BMI ≥ 25 kg/m^2^*N* = 10,537 (59.5)	Odds ratio	95% CI	Adj odds ratio	95% CI	*p*-value
Sex							
Women (ref)	4719 (66.6)	5163 (49.0)	1		1		
Men	2370 (33.4)	5374 (51.0)	2.073	1.947–2.206	2.035	1.899–2.181	**<0.001**

Age group (year)							
45–49 (ref)	1206 (17.0)	1321 (12.5)	1		1		
50–59	2309 (32.6)	3121 (29.6)	1.334	1.122–1.357	1.241	1.121–1.374	**<0.000**
60–69	2599 (36.7)	4285 (40.7)	1.505	1.373–1.650	1.400	1.249–1.570	**<0.001**
70–75	975 (13.8)	1810 (17.2)	1.695	1.518–1.892	1.547	1.325–1.807	**<0.001**

Education							
Primary school (ref)	751 (10.6)	1535 (14.6)	1		1		
Secondary school	1565 (22.1)	2818 (26.7)	0.881	0.792–0.980	1.028	0.916–1.154	0.640
Higher education	3764 (53.1)	4270 (40.5)	0.555	0.503–0.612	0.735	0.661–0.818	**<0.001**
Other	909 (12.8)	1704 (16.2)	0.917	0.814–1.033	1.003	0.885–1.137	0.963
Missing data	100 (1.4)	210 (2.0)	—	—	—	—	—

Occupation							
Working (ref)	4235 (59.7)	5607 (53.2)	1		1		
Sick leave/disability	154 (2.2)	353 (3.4)	1.731	1.427–2.101	1.517	1.234–1.864	**<0.001**
Retirement	2255 (31.8)	3899 (37.0)	1.306	1.223–1.394	1.104	0.996–1.224	0.060
Other	351 (5.0)	465 (4.4)	1.001	0.866–1.156	0.989	0.848–1.153	0.885
Missing	92 (1.3)	213 (2.0)	—	—	—	—	—

Marital status							
Single/living alone (ref)	946 (13.3)	1174 (11.1)	1		1		
Married/cohabitating	5084 (71.7)	7829 (74.3)	1.241	1.131–1.361	1.221	1.105–1.350	**<0.001**
Divorced/widowed	958 (13.5)	1329 (12.6)	1.118	0.992–1.259	1.177	1.036–1.336	**0.012**
Missing data	101 (1.4)	205 (1.9)	—	—	—	—	—

Smoking habits							
Never smoked (ref)	3771 (53.2)	4702 (44.6)	1		1		
Former smokers	2567 (36.2)	4718 (44.8)	1.474	1.382–1.572	1.385	1.291–1.485	**<0.001**
Current smokers	575 (8.1)	800 (7.6)	1.116	0.994–1.252	0.890	0.783–1.010	0.072
Missing data	176 (2.5)	317 (3.0)	—	—	—	—	—

Alcohol drinking frequency							
Never (ref)	236 (3.3)	463 (4.4)	1		1		
Once monthly or less	965 (13.6)	1774 (16.8)	0.937	0.786–1.117	0.874	0.685–1.113	0.275
2-3 times a month	1256 (17.7)	2008 (19.1)	0.815	0.686–0.968	0.728	0.570–0.931	**0.011**
Once weekly	1368 (19.3)	1984 (18.8)	0.739	0.623–0.877	0.604	0.473–0.773	**<0.001**
2-3 times a week	2363 (33.3)	3070 (29.1)	0.662	0.561–0.782	0.516	0.404–0.658	**<0.001**
≥4 times a week	679 (9.6)	823 (7.8)	0.618	0.513–0.745	0.440	0.340–0.571	**<0.001**
Missing data	222 (3.1)	415 (3.9)	—	—	—	—	—

Amount drinking/occasion							
1-2 glasses (ref)	4997 (70.5)	6371 (60.5)	1		1		
3-4 glasses	1432 (20.2)	2691 (25.2)	1.474	1.369–1.587	1.374	1.267–1.490	**<0.001**
≥5 glasses	191 (2.7)	652 (6.2)	2.677	2.269–3.159	2.027	1.699–2.420	**<0.001**
Missing/no drinking	469 (6.6)	823 (7.8)	—	—	—	—	—

*N* = number. Body mass index (BMI) was divided into <25 kg/m^2^ and ≥25 kg/m^2^. Logistic regression analysis was adjusted for all sociodemographic factors, smoking and alcohol habits, leisure time physical activity, and the regularity of meals. Values are presented as number and percentage or crude and adjusted (adj) odds ratio with a 95% confidence interval (CI). *p* value < 0.05 was considered statistically significant.

**Table 2:** Frequency of sociodemographic factors and smoking and alcohol habits in the EpiHealth cohort in relation to lean, normal-weight obesity, and overweight.

	Lean *N* = 2,995 (16.9)	Normal-weight obesity *N* = 3,941 (22.2)	Overweight *N* = 10,537 (59.4)
Sex			
Women	1921 (64.1)	2735 (69.4)	5163 (49.0)
Men	1074 (35.9)	1206 (30.6)	5374 (51.0)

Age group (year)			
45–49	726 (24.2)	471 (12.0)	1321 (12.5)
50–59	1072 (35.8)	1197 (30.4)	3121 (29.6)
60–69	913 (30.5)	1626 (41.3)	4285 (40.7)
70–75	284 (9.5)	647 (16.4)	1810 (17.2)

Education			
Primary school	241 (8.0)	494 (12.5)	1535 (14.6)
Secondary school	687 (22.9)	851 (21.6)	2818 (26.7)
Higher education	1693 (56.5)	1992 (50.5)	4270 (40.5)
Other	335 (11.2)	546 (13.9)	1704 (16.2)
Missing data	39 (1.3)	58 (1.5)	210 (2.0)

Occupation			
Working	2071 (69.1)	2103 (53.4)	5607 (53.9)
Sick leave/disability	58 (1.9)	89 (2.3)	353 (3.4)
Retirement	680 (22.7)	1502 (38.1)	3899 (37.0)
Other	150 (5.0)	192 (4.9)	465 (4.4)
Missing	36 (1.2)	55 (1.4)	213 (2.0)

Marital status			
Single/living alone	395 (13.2)	521 (13.2)	1174 (11.1)
Married/cohabitating	2154 (71.9)	2832 (71.9)	7829 (74.3)
Divorced/widowed	405 (13.5)	531 (13.5)	1239 (12.6)
Missing data	41 (1.4)	57 (1.4)	205 (1.9)

Smoking habits			
Never smoked	1694 (56.6)	2004 (50.9)	4702 (44.6)
Former smokers	977 (32.6)	1525 (38.7)	4718 (44.8)
Current smokers	250 (8.3)	313 (7.9)	800 (7.6)
Missing data	74 (2.5)	99 (2.5)	317 (3.0)

Drinking frequency			
Never	98 (3.3)	130 (3.3)	463 (4.4)
Once monthly or less	430 (14.4)	509 (12.9)	1774 (16.8)
2-3 times a month	554 (18.5)	681 (17.3)	2008 (19.1)
Once weekly	576 (19.2)	764 (19.4)	1984 (18.8)
2-3 times a week	1006 (33.6)	1309 (33.2)	3070 (29.1)
≥4 times a week	244 (8.1)	417 (10.6)	823 (7.8)
Missing data	74 (2.9)	99 (83.3)	317 (3.9)

Amount drinking/occasion			
1-2 glasses	2124 (70.9)	2764 (70.1)	6371 (60.5)
3-4 glasses	590 (19.7)	818 (20.8)	2691 (25.5)
≥5 glasses	86 (2.9)	100 (2.5)	652 (6.2)
Missing data	195 (6.5)	259 (6.6)	823 (7.8)

*N* = number. Values are presented as number and percentages by category. Lean is defined as a body mass index (BMI) of <25 kg/m^2,^ and a fat percentage in men of <20% and in women of <30%; normal-weight obesity (NWO) is defined as a BMI of <25 kg/m^2^ and a fat percentage in men of ≥20% and in women of ≥30%; and overweight is deined as a BMI of ≥25 kg/m^2^ [5, 6].

**Table 3:** Associations between lean, normal-weight obesity, and overweight with sociodemographic factors and smoking and alcohol habits in the EpiHealth cohort.

	Lean vs. NWO adj OR	95% CI	*p* value	Lean vs. overweight adj OR	95% CI	*p* value	NWO vs. overweight adj OR	95% CI	*p* value
Sex									
Women (ref)	1			1			1		
Men	0.664	**0.591–0.746**	**<0.001**	1.788	**1.621–1.973**	**<0.001**	2.290	**2.094–2.504**	**<0.001**

Age group (year)									
45–49 (ref)	1			1			1		
50–59	1.692	**1.452–1.971**	**<0.001**	1.531	**1.348–1.739**	**<0.001**	0.981	0.855–1.126	0.787
60–69	2.277	**1.905–2.723**	**<0.001**	2.026	**1.741–2.358**	**<0.001**	0.987	0.848–1.149	0.865
70–75	2.693	**2.070–3.503**	**<0.001**	2.596	**2.063–3.267**	**<0.001**	1.070	0.878–1.303	0.503

Education									
Primary school (ref)	1			1			1		
Secondary school	0.821	0.667–1.009	0.061	0.895	0.747–1.071	0.225	1.066	0.925–1.229	0.373
Higher education	0.771	**0.636–0.934**	**0.008**	0.608	**0.514–0.719**	**<0.001**	0.787	**0.691–0.896**	**<0.001**
Other	0.870	0.692–1.094	0.235	0.893	0.731–1.092	0.271	1.047	0.898–1.220	0.555
Missing data	—	—					—	—	

Occupation									
Working (ref)	1			1			1		
Sick leave/disability	1.097	0.750–1.602	0.634	1.799	**1.305–2.482**	**<0.001**	1.401	**1.066–1.843**	**0.016**
Retirement	1.561	**1.311–1.859**	**<0.001**	1.487	**1.272–1.737**	**<0.001**	0.955	0.842–1.084	0.474
Other	1.157	0.905–1.481	0.245	1.098	0.885–1.362	0.395	0.973	0.800–1.183	0.781
Missing	—	—					—	—	

Marital status									
Single/living alone (ref)	1			1			1		
Married/cohabitating	0.969	0.826–1.136	0.694	1.180	**1.025–1.360**	**0.022**	1.282	**1.141–1.391**	**<0.001**
Divorced/widowed	0.833	0.679–1.022	0.080	1.074	0.896–1.288	0.440	1.288	**1.505–2.376**	**0.002**
Missing data	—	—					—	—	

Smoking habits									
Never smoked (ref)	1			1			1		
Former smokers	1.116	0.995–1.252	0.061	1.479	**1.339–1.633**	**<0.001**	1.343	**1.232–1.465**	**<0.001**
Current smokers	0.814	0.662–1.000	0.050	0.836	**0.701–0.997**	**0.046**	0.964	0.823–1.129	0.647
Missing data	—	—					—	—	

Alcohol drinking frequency									
Never (ref)	1			1			1		
Once monthly or less	0.83	0.484–1.425	0.499	0.876	0.558–1.377	0.567	1.034	0.703–1.520	0.869
2-3 times a month	0.926	0.542–1.584	0.780	0.724	0.462–1.134	0.159	0.831	0.566–1.218	0.341
Once weekly	0.959	0.561–1.639	0.879	0.648	0.413–1.016	0.058	0.661	**0.451–0.970**	**0.034**
2-3 times a week	0.900	0.529–1.532	0.697	0.515	**0.330–0.805**	**0.004**	0.583	**0.399–0.852**	**0.005**
≥4 times a week	1.081	0.623–1.875	0.781	0.489	**0.307–0.778**	**0.003**	0.466	**0.314–0.691**	**<0.001**
Missing data	—	—					—	—	
Amount drinking/occasion									
1-2 glasses (ref)	1			1			1		
3-4 glasses	1.197	**1.048–1.368**	**0.008**	1.556	**1.387–1.739**	**<0.001**	1.260	**1.141–1.391**	**<0.001**
≥5 glasses	1.143	0.829–1.576	0.414	2.186	**1.705–2.799**	**<0.001**	1.889	**1.502–2.376**	**<0.001**
Missing/no drinking	—	—					—	—	

Lean is defined as a body mass index (BMI) of <25 kg/m^2^ and a fat percentage in men of <20% and in women of <30%; normal-weight obesity (NWO) is defined as a BMI of <25 kg/m^2^ and a fat percentage in men of ≥20% and in women of ≥30%; and overweight is defined as a of BMI ≥ 25 kg/m^2^ [5, 6]. Logistic regression analysis is adjusted for all sociodemographic factors, smoking and alcohol factors, leisure time physical activity, and the regularity of meals. Values are presented as adjusted (adj) odds ratio (OR) and 95% confidence interval (CI). *p* value < 0.05 was considered statistically significant.

**Table 4:** Associations between lower versus higher BMI and sociodemographic factors in relation to sex in the EpiHealth cohort.

	Women adj odds ratio	95% CI	Men adj odds ratio	95% CI	*p*_*i*_ value
Age group (year)					
45–49 (ref)	1		1		
50–59	1.383	1.214–1.574	1.049	0.886–1.244	**<0.001**
60–69	1.572	1.353–1.826	1.151	0.957–1.383	**<0.001**
70–75	1.864	1.513–2.297	1.160	0.915–1.469	**<0.001**

Occupation					
Working (ref)	1		1		
Sick leave/disability	1.807	1.416–2.307	1.016	0.690–1.495	**0.002**
Retirement	1.231	1.071–1.415	0.951	0.814–1.112	**<0.001**
Other	1.121	0.923–1.363	0.832	0.649–1.068	**0.040**
Missing	—	—	—	—	—

Low body mass index (BMI) was defined as < 25 kg/m^2^. A test for sex interaction was performed by adding a multiplicative variable to the full model of logistic regression analysis, adjusted for all sociodemographic factors, smoking and alcohol habits, leisure time physical activity, and the regularity of meals. Values are presented as an adjusted (adj) odds ratio and a 95% confidence interval (CI). A *p*_*i*_ value (*p* value for interaction) of <0.05 was considered statistically significant.

**Table 5:** Associations between normal-weight obesity versus overweight and sociodemographic factors in relation to sex in the EpiHealth cohort.

	Women adj odds ratio	95% CI	Men adj odds ratio	95% CI	*p*_*i*_ value
Age group (year)					
45–49 (ref)	1		1		
50–59	1.002	0.850–1.182	0.943	0.725–1.227	0.892
60–69	1.058	0.877–1.276	0.815	0.621–1.068	0.066
70–75	1.266	0.982–1.633	0.776	0.560–1.077	**0.001**

Occupation					
Working (ref)	1		1		
Sick leave/disability	1.746	1.271–2.399	0.714	0.421–1.211	**0.001**
Retirement	1.075	0.912–1.267	0.797	0.654–0.972	**<0.001**
Other	1.077	0.8471.369	0.807	0.574–1.133	0.136
Missing	—	—	—	—	—

Lean is defined as a body mass index (BMI) of <25 kg/m^2^ and a fat percentage in men of <20% and in women of <30%; normal-weight obesity is defined as a BMI of <25 kg/m^2^ and a fat percentage in men of ≥20% and in women of ≥30%; and overweight is defined as a BMI of ≥25 kg/m^2^ [5, 6]. A test for sex interaction was performed by adding a multiplicative variable to the full model of logistic regression analysis, adjusted for all sociodemographic factors, smoking and alcohol habits, physical activity, and the irregularity of meals. Values are presented as an adjusted (adj) odds ratio and a 95% confidence interval (CI). A *p*_*i*_-value (*p*-value for interaction) of <0.05 was considered statistically significant.

## Data Availability

The data that support the findings of this study are available from the steering group of EpiHealth but restrictions apply to the availability of these data, which were used under license for the current study and so they are not publicly available. Data are, however, available from the authors upon reasonable request and with permission from the steering group of EpiHealth.

## Abbreviations

Adj: Adjusted
BMI: Body mass index
CI: Confidence interval
NWO: Normal-weight obesity
OR: Odds ratio.

## References

[1] Kumanyika S. K., Obarzanek E., Stettler N. (2008). Population-based prevention of obesity. *Circulation*.

[2] Fox C. S., Massaro J. M., Hoffmann U. (2007). Abdominal visceral and subcutaneous adipose tissue compartments. *Circulation*.

[3] Addison O., Marcus R. L., Lastayo P. C., Ryan A. S. (2014). Intermuscular fat: a review of the consequenses and causes. *International Journal of Endocrinology*.

[4] Guclielmi V., Maresca L., D’Adamo M. (2014). Age-related different relationships between ectopic adipose tissues and measures of cenral obesity in sedentary subjects. *PLoS One*.

[5] De Lorenzo A., Del Gobbo V., Premrov M. G., Bigioni M., Galvano F., Di Renzo L. (2007). Normal-weight obese syndrome: early inflammation?. *The American Journal of Clinical Nutrition*.

[6] Männistö S., Harald K., Kontto J. (2014). Dietary and lifestyle characteristics associated with normal-weight obesity: the National FINRISK 2007 study. *British Journal of Nutrition*.

[7] Oliveros E., Somers V. K., Sochor O., Goel K., Lopez-Jimenez F. (2014). The concept of normal weight obesity. *Progress in Cardiovascular Diseases*.

[8] Karkhaneh M., Qorbani M., Mohajeri-Tehrani M. R., Hoseini S. (2017). Association of serum complement C3 with metabolic syndrome components in normal weight obese women. *Journal of Diabetes & Metabolic Disorders*.

[9] Musálek M., Pařízková J., Godina E. (2018). Poor skeletal robustness on lower extremities and weak lean mass development on upper arm and calf: normal weight obesity in middle-school-aged children (9 to 12). *Frontiers in Pediatrics*.

[10] Kwok A., Dordevic A. L., Paton G., Page M. J., Truby H. (2019). Effect of alcohol consumption on food energy intake: a systematic review and meta-analysis. *British Journal of Nutrition*.

[11] Hjartåker A., Lund E. (1998). Relationship between dietary habits, age, lifestyle, and socio-economic status among adult Norwegian women. The Norwegian women and cancer study. *European Journal of Clinical Nutrition*.

[12] Ruf T., Nagel G., Altenburg H.-P., Miller A. B., Thorand B. (2005). Food and nutrient intake, anthropometric measurements and smoking according to alcohol consumption in the EPIC Heidelberg study. *Annals of Nutrition and Metabolism*.

[13] Dušková M., Hruškovičová H., Šimůnková K., Stárka L., Pařízek A. (2014). The effects of smoking on steroid metabolism and fetal programming. *The Journal of Steroid Biochemistry and Molecular Biology*.

[14] Sjöholm K., Gripeteg L., Larsson I. (2016). Macronutrient and alcohol intake is associated with intermuscular adipose tissue in a randomly selected group of younger and older men and women. *Clinical Nutrition ESPEN*.

[15] Wadd S., Papadopoulos C. (2014). Drinking behavior and alcohol-related harm amongst older adults: analysis of existing UK datasets. *BMC Research Notes*.

[16] Krachler B., Eliasson M., Stenlund H., Johansson I., Hallmans G., Lindahl B. (2009). Population-wide changes in reported lifestyle are associated with redistribution of adipose tissue. *Scandinavian Journal of Public Health*.

[17] Stegmayr B., Lundberg V., Asplund K. (2003). The events registration and survey procedures in the Northern Sweden MONICA Project. *Scandinavian Journal of Public Health*.

[18] Ohlsson B., Manjer J. (2016). Physical inactivity during leisure time and irregular meals are associated with functional gastrointestinal complaints in middle-aged and elder subjects. *Scandinavian Journal of Gastroenterology*.

[19] Lind L., Elmståhl S., Bergman E. (2013). EpiHealth: a large population-based cohort study for investigation of gene–lifestyle interactions in the pathogenesis of common diseases. *European Journal of Epidemiology*.

[20] Norberg M., Lindvall K., Stenlund H., Lindahl B. (2010). The obesity epidemic slows among the middle-aged population in Sweden while the socioeconomic gap widens. *Global Health Action*.

[21] World Health Organization (2017). *Global Database on Body Mass Index*.

[22] Manjer J., Carlsson S., Elmstahl S. (2001). The Malmö diet and cancer study: representativity, cancer incidence and mortality in participants and non-participants. *European Journal of Cancer Prevention*.

[23] Berg C., Lappas G., Wolk A. (2009). Eating patterns and portion size associated with obesity in a Swedish population. *Appetite*.

[24] Richards M., Black S., Mishra G., Gale C. R., Deary I. J., Batty D. G. (2009). IQ in childhood and the metabolic syndrome in middle age: extended follow-up of the 1946 British Birth Cohort study. *Intelligence*.

[25] McCormak G. R., Friedenreich C., McLaren L., Potestio M., Sandalack B., Csizmadi I. (2017). Interactions between neighbourhod urban form and socioeconomic status and their associations with anthropometric measurements in Canadian adults. *Journal of Environmental and Public Health*.

[26] Strandhagen E., Berg C., Lissner L. (2010). Selection bias in a population survey with registry linkage: potential effect on socioeconomic gradient in cardiovascular risk. *European Journal of Epidemiology*.

[27] Cornelis M. C., Chiuve S. E., Glymour M. M. (2014). Bachelors, divorcees, and widowers: does marriage protect men from type 2 diabetes?. *PLoS One*.

[28] Berlin I. (2009). Endocrine and metabolic effects of smoking cessation. *Current Medical Research and Opinion*.

[29] Ishikawa M., Yokoyama T., Murayama N. (2017). Alcohol energy intake is related to low body mass index in Japanese older adults: data from the 2010-2011 national health and nutrition survey. *The Journal of Nutrition, Health & Aging*.

[30] Sydén L., Sidorchuk A., Mäkelä P., Landberg J. (2017). The contribution of alcohol use and other behavioural, material and social factors to socio-economic differences in alcohol-related disorders in a Swedish cohort. *Addiction*.

[31] Patel P., Abate N. (2013). Body fat distribution and insulin resistance. *Nutrients*.

[32] Grodner M., Long Roth S., Walkingshaw B. C. (2011). *Nutritional Foundations and Clinical Applications: A Nursing Approach*.

[33] Alwachi S. N., Khazaal F. A., Yenzeel J. H., Karim N. (2014). Waist hip ratio as predictors of obesity types in postmenopausal Iraq women. *European Journal of Public Health*.

[34] Ley C. J., Lees B., Stevenson J. C. (1992). Sex- and menopause-associated changes in body-fat distribution. *The American Journal of Clinical Nutrition*.

[35] Tognon G., Malmros V., Freyer E., Bosaeus I., Mehlig K. (2015). Are segmental MF-BIA scales able to reliably assess fat mass and lean soft tissue in an elderly Swedish population?. *Experimental Gerontology*.

